# Integrated transcriptomic and immune analysis reveals distinct mitochondrial and immune signature in COVID-19 ARDS requiring invasive mechanical ventilation

**DOI:** 10.3389/fmed.2026.1856132

**Published:** 2026-07-08

**Authors:** Deepa B. Gotur, Diksha M. Gowda, Decha Pinkaew, Aijun Zhang, Spencer Hankins, Shaefali Rodgers, Yitian Xu, Mohi U. Syed, Tejaswini Reddy, Hong Zhao, Junjun Zheng, Rodney J. Folz, Eleftherios Mylonakis, Dale J. Hamilton

**Affiliations:** 1 Division of Pulmonary, Critical Care, and Sleep Medicine, Houston Methodist Hospital, Houston, TX, United States; 2 Charles W. Duncan Jr. Department of Medicine, Houston Methodist, Houston, TX, United States; 3 Center for Bioenergetics Research, Houston Methodist Research Institute, Houston, TX, United States; 4 Immunomonitoring Core, Houston Methodist Neal Cancer Center, Houston, TX, United States; 5 Center for Inflammation and Infectious Disease, Houston Methodist Research Institute, Houston, TX, United States; 6 Department of Systems Medicine and Bioengineering, Houston Methodist Cancer Center, Houston, TX, United States

**Keywords:** ARDS, biomarkers, COVID-19, cytokine, immune exhaustion, mechanical ventilation, mitochondrial dysfunction, ventilation

## Abstract

**Introduction:**

Severe COVID-19–related acute respiratory distress syndrome (ARDS) often progresses to respiratory failure requiring invasive mechanical ventilation (IMV). Although several biomarkers (e.g., CRP, suPAR, Ang-2) have been explored to predict COVID-19 disease severity, validated early-presentation biomarkers that specifically prognosticate the need for IMV and mechanistic pathways in ARDS remain limited.

**Research question:**

What transcriptomic and immune signatures distinguish IMV from non-IMV in patients with COVID-19 ARDS, and how do these molecular changes contribute to disease severity?

**Methods:**

Patients (*n* = 36) with SARS-CoV-2 infection requiring oxygen support were enrolled in this prospective study. Blood samples were collected at baseline, day 4, and day 8 from patients on IMV (*n* = 11) and non-IMV (*n* = 25). Transcriptomic profiles of peripheral blood mononuclear cells were assessed using RNA-sequencing, followed by gene ontology and KEGG pathway enrichment. Plasma cytokines and chemokines were measured using multiplex immunoassays, peripheral immune cell endotypes were characterized by mass cytometry, and neutrophil extracellular trap (NET) formation was evaluated by kinetic live-cell imaging.

**Results:**

Clinically, IMV patients had significantly longer hospital (43.0 vs. 19.5 days, *p* = 0.001) and ICU stays (39.6 vs. 9.1 days, *p* < 0.01) and higher 28-day mortality (45.5% vs. 12.0%; aOR = 8.78, *p* = 0.04). Transcriptomic profiling identified 36 DEGs at day 4 and 21 at day 8 in patients on IMV, characterized by downregulation of mitochondrial gene MTARC2 and anticoagulant regulator TFPI, and upregulation of stress-response genes (RNF165, KCNMA1, and AC245014.3) (*p* < 0.05). Plasma IL-6, IL-8, and IL-10 were elevated in IMV group, with IL-10 showing the strongest predictive value (AUC 0.74, *p* = 0.0002). Immune profiling revealed higher percentage of CD57^−^ NK cells at baseline in non-IMV group. NET formation did not differ between groups.

**Conclusion:**

Patients requiring IMV demonstrated distinct transcriptomic and immune profiles associated with severe COVID-19. These findings identify candidate biomarkers and pathways associated with disease severity; however, the results are exploratory and require validation in larger cohorts and functional studies.

## 1 Introduction

Severe acute respiratory syndrome from SARS-CoV-2, a strain of RNA coronavirus that caused the COVID-19 pandemic, endangered the lives of millions globally (1). Among hospitalized patients, acute respiratory distress syndrome (ARDS) emerged as the most fatal complication, characterized by severe impairment of gas exchange due to intense pulmonary inflammation, resulting in alveolar–capillary leakage, surfactant depletion and dysfunction, and ultimately pulmonary fibrosis (2). This process is largely driven by a catastrophic hyperinflammatory response, termed the cytokine storm. The resulting inflammation is associated with profound oxidative stress and triggers expression of inducible nitric oxide synthase (iNOS), leading to excessive production of reactive oxygen species (ROS) and reactive nitrogen species (RNS). Together, these processes impair mitochondrial function and exacerbate intracellular hypoxia (3). Emerging evidence points to distinct mitochondrial alterations in COVID-19 ARDS, but comprehensive transcriptomic profiling to define disease progression to respiratory failure has not yet been performed (4).

Neutrophil extracellular traps (NETs) have also been implicated in the pathogenesis of severe COVID-19 through their proposed roles in inflammation, endothelial injury, immunothrombosis, and ARDS progression (5). Excessive NET formation has been associated with disease severity and adverse outcomes in COVID-19, supporting investigation of NETosis as a potential contributor to respiratory failure requiring invasive mechanical ventilation (IMV).

Clinically, approximately one in five patients with COVID-19 develops respiratory distress requiring supplemental oxygen or IMV (6), and among those admitted to the ICU, nearly one in three die (7). The ability to identify reliable prognostic biomarkers is therefore critical for recognizing patients at increased risk of respiratory decompensation and progression to IMV.

We hypothesized that mitochondrial dysfunction represents a defining feature of severe COVID-19 ARDS, and that cytokine release and immune exhaustion are interconnected consequences of impaired mitochondrial activity. To test this hypothesis, we evaluated transcriptomic signatures of peripheral blood mononuclear cells (PBMCs), plasma cytokine levels, and immune cell phenotypes in patients with COVID-19, comparing those who required IMV with those who did not.

## 2 Materials and methods

This prospective observational study was conducted between September 10, 2020, and March 1, 2022, at Houston Methodist Hospital. The study was approved by the Institutional Review Board, and informed consent was obtained from all participants or their legal representatives. Adults (≥18 years) with PCR-confirmed SARS-CoV-2 infection requiring supplemental oxygen were enrolled and stratified according to the WHO clinical progression scale into non-mechanically ventilated (scores 5–6) and mechanically ventilated (scores 7–9) groups.

Fresh whole blood was collected in EDTA tubes at baseline, Day 4, and Day 8, with a maximum total collection volume of 180 mL. PBMCs were isolated within 24 h of collection and stored at −150 °C for subsequent RNA sequencing and mass cytometry (8). Plasma samples were analyzed for cytokine and chemokine levels and used to stimulate healthy donor neutrophils for quantification and visualization of NETosis. All patient data and biospecimens were de-identified, and researchers performing the analyses were blinded to patient group assignments. Survivors were contacted by telephone to assess 28-day mortality outcome.

Sample Size and Power Calculation: Initially, we calculated that a minimum of 20 patients per group (IMV vs. non-IMV) would provide 80% power to detect a 2-fold or greater difference between groups at a significance level of 0.05. During the study period, we enrolled a total of 36 patients, of whom 11 (30%) required IMV and 25 did not. The overall number of patients requiring IMV was lower than initially projected, likely reflecting declining intubation rates with the waning of the COVID-19 Delta variant, widespread vaccination, and advances in supportive care. Nevertheless, because 30% of our cohort (11/36) required IMV, we were able to proceed with comparative analyses between the two groups.

Statistical Methods: We reported means and standard deviations (SD) for continuous variables, and proportions for categorical variables. Histograms and the Shapiro–Wilk test were used to assess the normality of continuous variables and outcomes. Student’s *t*-test was used to assess mean differences between continuous variables. The chi-squared test (χ^2^) or Fisher’s exact test, as appropriate, was used to evaluate differences between categorical variables. The primary variable of interest was the IMV status: patients who were on IMV (1) vs. those who were not (0). The primary outcomes included 28-day mortality (1 = Died, 0 = Alive), 30-day readmission (1 = Readmitted, 0 = Not readmitted), hospital length of stay, and ICU length of stay. For binary outcomes, both univariate and multivariate generalized logistic regression models were used. For continuous outcomes, such as hospital and ICU length of stay, generalized linear regression models were employed. All models were adjusted for demographics (age, gender, and BMI), comorbidities, Charlson Comorbidity Index (CCI), and medications to reduce bias. A *p*-value <0.05 was considered statistically significant. We reported adjusted odds ratios (aOR) with 95% confidence intervals (CI), as well as coefficient estimates (Δ*β*). All statistical analyses were performed using R version 4.5.1.

### 2.1 RNAseq and gene expression analysis

PBMCs were isolated from blood samples collected at baseline, day 4, day 8, and, where applicable, on the day of IMV initiation and liberation. Frozen cell pellets were then submitted to Azenta Life Sciences/GENEWIZ (South Plainfield, NJ) for Whole-transcriptome RNA sequencing (Tracking #30-696137733). RNA extraction from frozen cell pellets, library preparation using poly-A selection for mRNA enrichment, and sequencing on an Illumina platform with paired-end 2 × 150 bp configuration targeting 20–30 million reads per sample were performed by GENEWIZ. RNA integrity and library quality were assessed by GENEWIZ using Agilent TapeStation, Qubit fluorometric quantification, and qPCR before sequencing. Samples with inadequate RNA integrity or sequencing quality metrics were excluded from downstream analyses. A total of 95 samples from 11 IMV and 25 non-IMV patients across available timepoints were included in the final analysis. A comparison of gene expressions between the groups of samples was performed using DESeq2. The Wald test was applied to generate *p*-values and log2 fold changes. Genes with an adjusted *p*-value <0.05 and absolute log2 fold change >1 were classified as differentially expressed genes (DEGs). Gene ontology (GO) analysis was performed using g:Profiler (version e107_eg54_p17_bf42210, database updated 15/09/2022) to visualize the enrichment information of the significant DEGs. *Homo sapiens* was chosen as a specific organism for analysis. GO molecular function (GO:MF), GO cellular component (GO:CC), and GO biological process (GO:BP) categories were used as Gene Ontology data sources. Pathway analysis used KEGG, Reactome, and WikiPathways databases. Analyses were restricted to annotated genes with significance thresholds set using g:SCS multiple testing correction method at *α* = 0.05. DEGs were further analyzed using gene set enrichment analysis via ShinyGO (9). Significant DEGs were imported into the ShinyGO V0.77 tool to perform KEGG pathway analysis and obtain a visual graph of KEGG pathways. When submitting the list of gene targets in the Gene Official Symbol format, the selected matching species were human, and the FDR cutoff was set to 0.05 (*p* ≤ 0.05). After running the analysis, the top five KEGG pathways that matched the conditions were sorted by number of genes and visualized in the graphs.

The GOnet web application was used to identify biological functions of significant DEGs at Day 4 (10). The following analysis parameters were used: species: *homo sapiens*; input proteins: 36; GO namespace: biological process; analysis type: GO-term enrichment; *q*-value threshold of 0.05. Enrichment analysis of significant DEGs at Day 4 was identified in DisGeNET, a database of gene-disease associations (11). All genes in the genome were used as the enrichment background. Terms with *p* < 0.01, a minimum count of 3, and an enrichment factor > 1.5 (the enrichment factor is the ratio between the observed counts and the counts expected by chance) were collected and grouped into clusters based on their membership similarities. The overlap and unique significant DEGs from Day 4 and Day 8 in the IMV group versus the non-IMV group were analyzed as Venn diagram using jvenn (12).

### 2.2 Cytokine and chemokine assay

Plasma concentrations of 38 analytes were measured by a Luminex multiplex immunoassay (Human Cytokine/Chemokine Magnetic Bead Panel, HCYTMAG-60 K-PX41, EMD Millipore, Burlington, MA). The panel included EGF, eotaxin, FGF-2, Flt-3L, fractalkine, G-CSF, GM-CSF, GRO, IFN-*α*2, IFN-*γ*, IL-1α, IL-1*β*, IL-1RA, IL-2, IL-3, IL-4, IL-5, IL-6, IL-7, IL-8, IL-9, IL-10, IL-12p40, IL-12p70, IL-13, IL-15, IL-17A, IP-10, MCP-1, MCP-3, MDC, sCD40L, MIP-1α, MIP-1*β*, TGF-α, TNF-α, TNF-*β*, and VEGF. Baseline, Day 4, Day 8, and/or Day-of-discharge samples were included in the longitudinal analysis, comprising 11 IMV and 25 non-IMV patients. For patients discharged before the scheduled Day 4 or Day 8 collection time points, the sample obtained closest to the day of discharge was assigned to the corresponding Day 4 or Day 8 time point for longitudinal analysis. Healthy donor controls were analyzed in parallel for cytokine/chemokine profiles. Outliers were identified with Grubbs’ test and excluded. Analyte concentrations were assessed using mixed-effects models with patient group (IMV vs. non-IMV) and time point (baseline, Day 4, Day 8) as fixed factors, without assuming sphericity. Significant interaction effects were further evaluated with Tukey’s *post hoc* tests. Diagnostic performance of differentially expressed analytes was assessed by receiver operating characteristic (ROC) curve analysis, with area under the curve (AUC) used to estimate discrimination between IMV and non-IMV patients. Statistical analyses were performed using GraphPad Prism (version 10.0.3; GraphPad Software, Boston, MA), with significance set at *α* = 0.05.

### 2.3 NETosis assay

Peripheral neutrophil extracellular trap (NET) formation was quantified in patient’s plasma by measuring myeloperoxidase (MPO)–DNA complexes using a commercial ELISA kit (11774425001; Roche, Mannheim, Germany). Age-matched samples from 10 IMV and 10 non-IMV patients were included in the analysis. To further evaluate NETosis, neutrophils isolated from healthy donors were incubated with plasma from either healthy controls or patients with COVID-19 at different disease stages. NET release was assessed by time-lapse imaging using Incucyte^®^ Netosis Assay (Sartorius, Göttingen, Germany) (13).

### 2.4 Mass cytometry

Cytometry by time-of-flight (CyTOF^®^, Fluidigm) was performed for comprehensive immune profiling using patients’ PBMCs at an institutional core facility. A total of 30 PBMC samples from 10 patients (6 IMV and 4 non-IMV patients), collected at multiple time points, were analyzed using two CyTOF panels: a 36-marker myeloid panel (M-panel) and a 36-marker T cell panel (T-panel). Prior to staining, cryopreserved PBMCs were thawed and assessed for viability. Standardized quality control procedures were applied, including normalization using calibration beads, exclusion of debris and dead cells, and doublet discrimination before downstream analyses. Samples with inadequate viability or poor event quality were excluded. Data preprocessing and gating strategies were performed uniformly across all samples to minimize batch-related variability and ensure consistency in immune cell population identification. We analyzed differential abundance of immune cell populations between IMV and non-IMV groups at each time point using unpaired *t*-test or Welch’s *t*-test with Holm-Šídák correction for multiple comparisons. Within-group differences were evaluated using one-way mixed-effects models without assuming sphericity, followed by Tukey’s *post hoc* tests. Statistical analyses were performed using GraphPad Prism, with significance set at *α* = 0.05.

To address multiple testing concerns, corrections for multiple comparisons were applied separately for the transcriptomic, cytokine, and immunophenotyping analyses. In the RNA-seq analysis, differential gene expression was assessed using DESeq2 with Benjamini–Hochberg false discovery rate (FDR) adjustment, and genes with an adjusted *p*-value <0.05 were considered significant. For pathway enrichment analyses, significance thresholds were controlled using g:SCS correction in g:Profiler and FDR-adjusted *p*-values in ShinyGO. In the cytokine and chemokine analyses, mixed-effects models were followed by Tukey’s *post hoc* tests to account for multiple pairwise comparisons across groups and time points, and adjusted *p*-values are reported where applicable. For CyTOF immunophenotyping, differential immune cell abundance between IMV and non-IMV groups was evaluated using unpaired *t*-tests or Welch’s *t*-tests with Holm–Šídák correction for multiple comparisons, while longitudinal within-group analyses used mixed-effects models followed by Tukey’s *post hoc* testing. Adjusted *p*-values were used to determine statistical significance throughout these analyses.

## 3 Results

Our study population consisted of 36 patients, of whom 11 required IMV. The mean age was similar between the two groups (65 vs. 62; *p* = 0.49). 64% percent of the patients were male, with a mean BMI of 31 in the intubation group. Additionally, 18% of patients in the intubation group were ex-smokers. The mean daily steroid equivalent dosage was 9 mg in the IMV patients versus 34 mg in the non-IMV patients (*p* = 0.01). Table 1 describes the baseline characteristics of the study population.

**Table 1:** Baseline characteristics of patients with COVID-19 ARDS.

Variables	non-IMV	IMV	*P*-value
*N*	**25**	**11**	
Age (mean (SD))	61.92 (11.41)	64.73 (10.46)	0.49
Gender – Male	15 (60.0)	7 (63.6)	1.00
BMI (mean (SD))	31.22 (6.72)	31.27 (6.68)	0.98
Current smoker *N* (%)	1 (4.0)	0 (0.0)	1.00
Ex-smoker *N* (%)	7 (28.0)	2 (18.2)	0.84
CCI (mean (SD))	1.80 (1.89)	2.18 (1.08)	0.54
Individual comorbid conditions *N* (%)			
Hypertension	14 (56.0)	6 (54.5)	1.00
Diabetes mellitus II	13 (52.0)	4 (36.4)	0.62
Coronary artery disease	3 (12.0)	0 (0.0)	0.59
CHF – Heart failure	3 (12.0)	1 (9.1)	1.00
Hypersensitivity lung disease	7 (28.0)	1 (9.1)	0.41
Cerebrovascular accident	2 (8.0)	1 (9.1)	1.00
Cirrhosis	2 (8.0)	0 (0.0)	0.86
CKD – chronic kidney disease	5 (20.0)	0 (0.0)	0.28
ESRD – End-stage renal disease	7 (28.0)	2 (18.2)	0.84
COPD – Chronic obstructive pulmonary disease	1 (4.0)	0 (0.0)	1.00
Asthma	1 (4.0)	1 (9.1)	1.00
Active malignancy	1 (4.0)	2 (18.2)	0.45
Medications *N* (%)			
ACE-I – Angiotensin-converting enzyme	1 (4.0)	2 (18.2)	0.45
ARB	4 (16.0)	1 (9.1)	0.98
Remdesivir	14 (56.0)	4 (36.4)	0.47
Hydroxychloroquine	2 (8.0)	0 (0.0)	0.86
Tocilizumab	1 (4.0)	2 (18.2)	0.45
Steroids	24 (96.0)	10 (90.9)	1
Convalescent – plasma	0 (0.0)	1 (9.1)	0.669
Antibiotics	15 (60.0)	7 (63.6)	1
Steroid – dose - per-day (mean (SD))	33.93 (25.97)	9.17 (5.78)	<0.05
Outcomes			
28th day mortality – *N* (%)	3 (12.0)	5 (45.5)	0.07
30- day readmission – *N* (%)	3 (12.0)	1 (9.1)	1
Hospital – length-of-stay (mean (SD))	19.52 (12.44)	43.00 (27.41)	0.001
ICU – length-of-stay (mean (SD))	9.12 (5.12)	39.55 (27.75)	<0.001

Our study population consisted of 36 patients, of whom 11 belonged to the IMV group and 25 to the non-IMV group. IMV: Invasive Mechanical Ventilation, non-IMV: non-IMV: not on Invasive Mechanical Ventilation, N: total number of subjects, SD: Standard Deviation, BMI: Body Mass Index, CCI: Charlson Co-morbidity Index, CHF: Congestive Heart Failure, CKD: Chronic Kidney Disease, ESRD: End Stage Renal Disease, COPD: Chronic Obstructive Pulmonary Disease, ACE-I: Angiotensin Converting Enzyme Inhibitor, ARB: Angiotensin Receptor Blocker, ICU: Intensive Care Unit.

45.5% of patients in the IMV group died after the 28th day, compared to 12.0% of patients who were in the non-IMV group. Additionally, the multivariate model showed that IMV group patients were eight times more likely to die by the 28th day compared to non-IMV group patients (aOR = 8.78; 95% CI [1.09–70.85]; *p* = 0.04). Table 2 presents the full model.

**Table 2:** Outcome- 28th day mortality [Yes = 1 vs. No = 0].

Outcome- 28-day mortality – [Yes = 1 vs. No = 0]		
Variables	Adjusted- odds ratio (95% CI)	*P*-value
Group – non-IMV	Ref	Ref
IMV	**8.78 [1.09–70.85]**	**0.04**
Hypertension [Yes vs. No]	0.32 [0.03–3.05]	0.32
Diabetes mellitus II [Yes vs. No]	0.79 [0.1–6.02]	0.82
Congestive heart failure [Yes vs. No]	1.31 [0.08–21.33]	0.85
Hypersensitivity lung disease [Yes vs. No]	0.86 [0.04–17.36]	0.92
ESRD – End-stage renal disease [Yes vs. No]	3.14 [0.3–32.57]	0.34
ARB [Yes vs. No]	2.98 [0.08–106.99]	0.55
Remdesivir [Yes vs. No]	1 [0.09–10.68]	1
Tocilizumab [Yes vs. No]	0.85 [0.02–37.15]	0.93
BMI	0.98 [0.83–1.16]	0.8

The multivariable analysis showed that patients who required IMV had an approximately ninefold higher likelihood of dying within 28 days compared with those who did not require IMV (adjusted odds ratio [aOR] = 8.78; 95% CI, 1.09–70.85; *p* = 0.04).

9% of patients in the IMV group were readmitted within 30 days, compared to 12% of patients in the non-IMV group. The multivariate model showed that IMV group patients were twice as likely to be readmitted within 30 days; however, this difference was not statistically significant (aOR = 2.77; 95% CI [0.09–86.38]; *p* = 0.56).

The mean length of hospital stay (in days) was higher in patients in IMV group compared to those in the non-IMV group (43.0 vs. 19.5; Δ*β* = 19.67; 95% CI [6.8–32.6]; *p* = 0.01). Table 3 presents the full model. The mean ICU length of stay (in days) was also higher in patients in IMV group compared to those in the non-IMV group (39.6 vs. 9.1; Δ*β* = 22.4; 95% CI [9.87–34.99]; *p* < 0.001). Table 4 presents the full model.

**Table 3:** Outcome- hospital length of stay in days.

Outcome = Hospital length of stay				
Variables	Δ*β*	Lower CI	Upper CI	Pr(>|t|)
Group – non-IMV	Ref	Ref	Ref	Ref
IMV	19.67	6.76	32.58	0.01
Age	−0.27	−0.83	0.28	0.35
Gender – Male [Female = Reference]	−3.46	−15.42	8.5	0.58
BMI	−0.91	−1.85	0.03	0.07
CCI	0.54	−2.86	3.94	0.76
ARB (Yes vs. No)	3.42	−13	19.84	0.69
Remdesivir (Yes vs. No)	10.92	−1.46	23.31	0.1
Hydroxychloroquine (Yes vs. No)	−10.79	−38.98	17.4	0.46
Tocilizumab (Yes vs. No)	1.64	−21.3	24.59	0.89
Steroids (Yes vs. No)	1.11	−21.63	23.86	0.92
Convalescent plasma	64.69	29.56	99.81	<0.001

**Table 4:** Outcome- ICU length of stay in days.

Outcome- ICU- length of stay				
Variables	Δ*β*	Lower CI	Upper CI	Pr(>|t|)
Group – non-IMV	Ref	Ref	Ref	Ref
IMV	22.43	9.87	34.99	<0.001
Age	−0.19	−0.7	0.32	0.47
Gender – Male [Female = Reference]	−3.49	−15.75	8.76	0.58
BMI	−0.82	−1.92	0.29	0.17
CCI	1.02	−2.68	4.73	0.6
ARB (Yes vs. No)	16.63	−3.04	36.31	0.12
Remdesivir (Yes vs. No)	−2.86	−14.97	9.25	0.65
Hydroxychloroquine (Yes vs. No)	−7.91	−33.16	17.33	0.55
Tocilizumab (Yes vs. No)	8.23	−13.34	29.8	0.47
Steroids (Yes vs. No)	2.6	−24.74	29.93	0.85
Convalescent plasma	81.12	49.95	112.29	<0.001

### 3.1 Transcriptomic profiling and analysis of PBMCs

**Figure 1:**
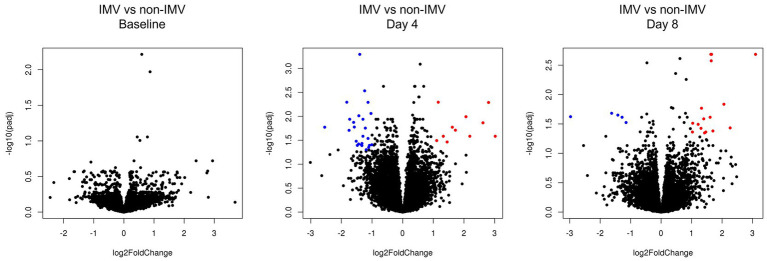
Differentially expressed genes (DEGs) in IMV versus non-IMV COVID-19 ARDS patients. Volcano plots show global transcriptional changes in PBMC at baseline, day 4, and day 8. Each dot represents a single gene, with the x-axis showing log2 fold change and the y-axis showing –log10 adjusted *p*-value. Genes with adjusted *p* < 0.05 and log2 fold change > 1 are highlighted in red (upregulated), and those with *p*-value less than 0.05 and a log2 fold change less than −1 are highlighted in green (downregulated). At baseline, no DEGs were detected. At Day 4, 36 DEGs were identified (25 downregulated, 11 upregulated), and at Day 8, 21 DEGs were identified (5 downregulated, 16 upregulated). Numbers in the lower corners indicate counts of downregulated (left) and upregulated (right) genes at each time point.

To identify underlying molecular mechanisms of disease severity in patients with COVID-19, we isolated PBMCs and performed RNAseq and gene expression analysis, comparing transcriptomic profiles of PBMCs from the IMV group with those from the non-IMV group. Global transcriptional changes are visualized in Figure 1, where each data point in the volcano plot represents a gene, with log2-fold change on the x-axis and the -log10 adjusted *p*-value on the y-axis. Genes with adjusted *p* < 0.05 and log2-fold >1 (red) were considered upregulated, while those with adjusted *p* < 0.05 and log2-fold change <−1 (blue) were considered downregulated. When comparing patients in IMV with the non-IMV group across all time points, a total of 0, 36, and 21 genes were differentially expressed in the IMV group compared with the non-IMV group at baseline, Day 4, and Day 8, respectively. These correspond to 0.00, 0.20, and 0.11% of all genes assessed (18,222, 18,222, and 18,365 genes, respectively) (Figure 1). These DEGs comprised 0, 11, and 16 upregulated genes (accounting for 0.00, 30.56, and 76.19% of all significant DEGs) and 0, 25, and 5 downregulated genes (accounting for 0.00, 69.44, and 23.81% of all significant DEGs) at baseline, Day 4, and Day 8, respectively.

To explore the biological significance of these changes, we next performed pathway enrichment analysis. At Day 4, gene ontology molecular function (GO:MF) analysis identified enrichment of a single term (vascular endothelial growth factor receptor activity). In contrast, 16 terms were enriched in gene ontology biological processes (GO:BP). Many related to cellular response to stimuli and regulation of multicellular processes, including negative regulation of response to external stimuli, negative regulation of chronic inflammatory response, and response to carbon monoxide (Supplementary Figure 1a). KEGG pathway analysis further demonstrated enrichment of Renin secretion and Rap1 signaling pathways in PBMCs from IMV group compared to non-IMV (Supplementary Figure 1b). DisGeNET enrichment analysis linked differentially expressed genes to clinical phenotypes such as anoxia, essential thrombocythemia, neointima formation, visceral pain, and bronchopulmonary dysplasia (Supplementary Figure 1c).

**Figure 2:**
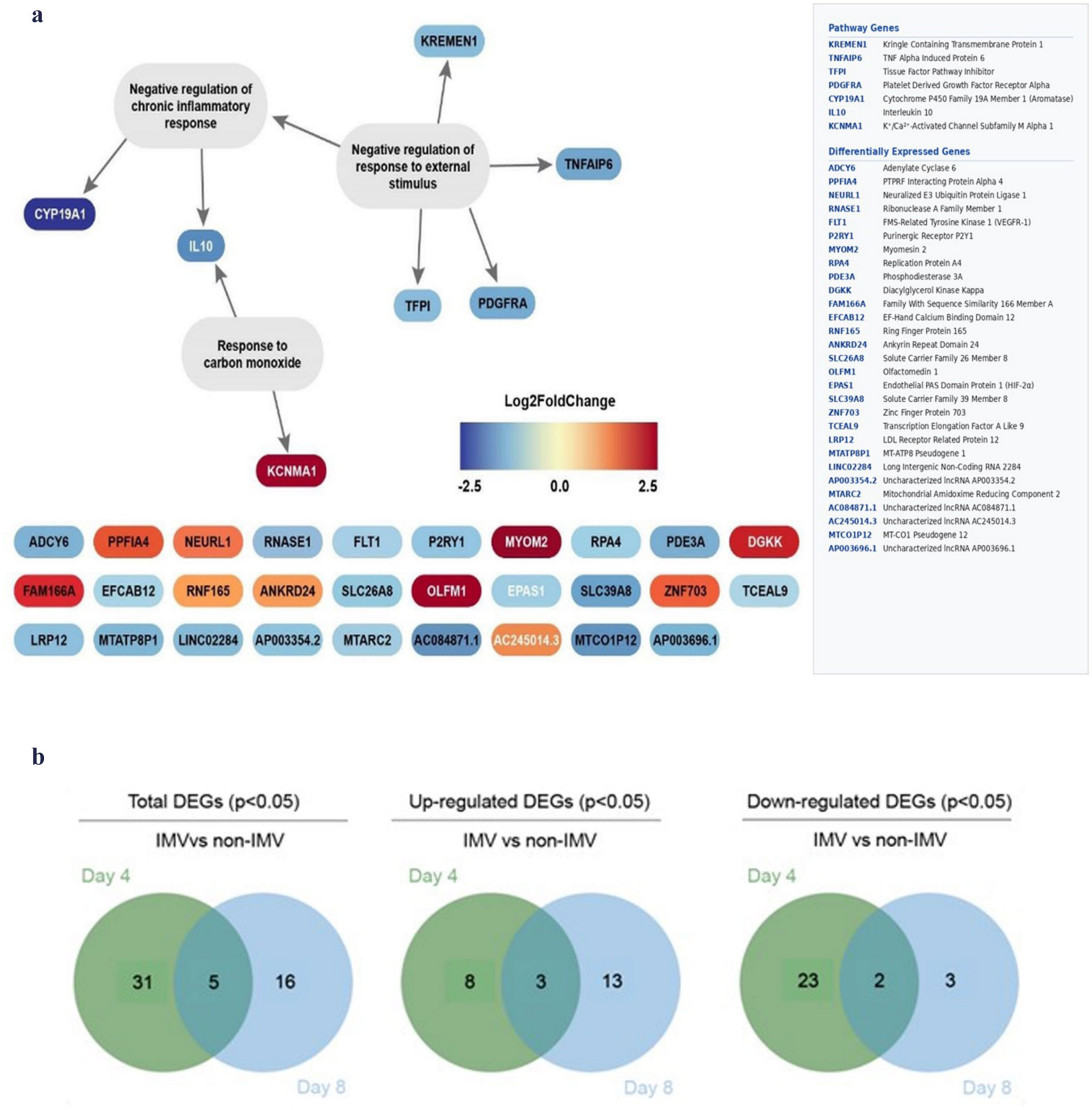
**(a)** Functional enrichment analysis of differentially expressed genes (DEGs) in IMV versus non-IMV groups at Day 4. Gene-term interaction networks highlight biological processes enriched among DEGs. Downregulated genes (green) included TFPI, PDGFRA, TNFAIP6, and KREMEN1, associated with negative regulation of response to external stimulus. Downregulation of IL-10 and CYP19A1 was linked to negative regulation of chronic inflammatory response. Response to carbon monoxide involved upregulation of KCNMA1 (red) and downregulation of IL-10. Heatmap bar indicates log2 fold change values. **(b)** Overlap of DEGs between Day 4 and Day 8 in IMV versus non-IMV groups. Venn diagrams show the number of total, upregulated, and downregulated DEGs (adjusted *p* < 0.05) identified at Day 4 (green) and Day 8 (blue). Five DEGs were shared between time points: three upregulated (RNF165, KCNMA1, AC245014.3) and two downregulated (TFPI, MTARC2).

We identified biological functions of significant DEGs at Day 4 to evaluate gene-term interactions. As shown in Figure 2a, the negative regulation of response to external stimulus pathway involved the downregulation of TFPI, PDGFRA, TNFAIP6, and KREMEN1 expression. Negative regulation of the chronic inflammatory response pathway included the downregulation of IL-10 and CYP19A1 expression, whereas response to carbon monoxide involved the up-regulation of KCNMA1 and the downregulation of IL-10 expression. We further identified uniquely significant DEGs from Day 4 and Day 8 of patients on IMV compared with non-IMV group as shown by Venn diagram (Figure 2b). Five DEGs were shared across both time points: three were upregulated (RNF165, KCNMA1, and AC245014.3) and two were downregulated (TFPI and MTARC2) in IMV patients compared with non-IMV patients (Table 5).

**Table 5:** List of the overlap and unique significant DEGs from day 4 and day 8 of IMV patients compared with non-IMV patients using Venn diagram.

Expression pattern	Gene	Description
Downregulated	TFPI	Tissue factor pathway inhibitor
	MTARC2	Mitochondrial amidoxime reducing component 2
Up regulated	RNF165	Ring finger protein 165
	KCNMA1	Potassium calcium-activated channel subfamily M alpha 1
	AC245014.3	Novel transcript with unknown function

### 3.2 Immunoassays of peripheral cytokines

**Figure 3:**
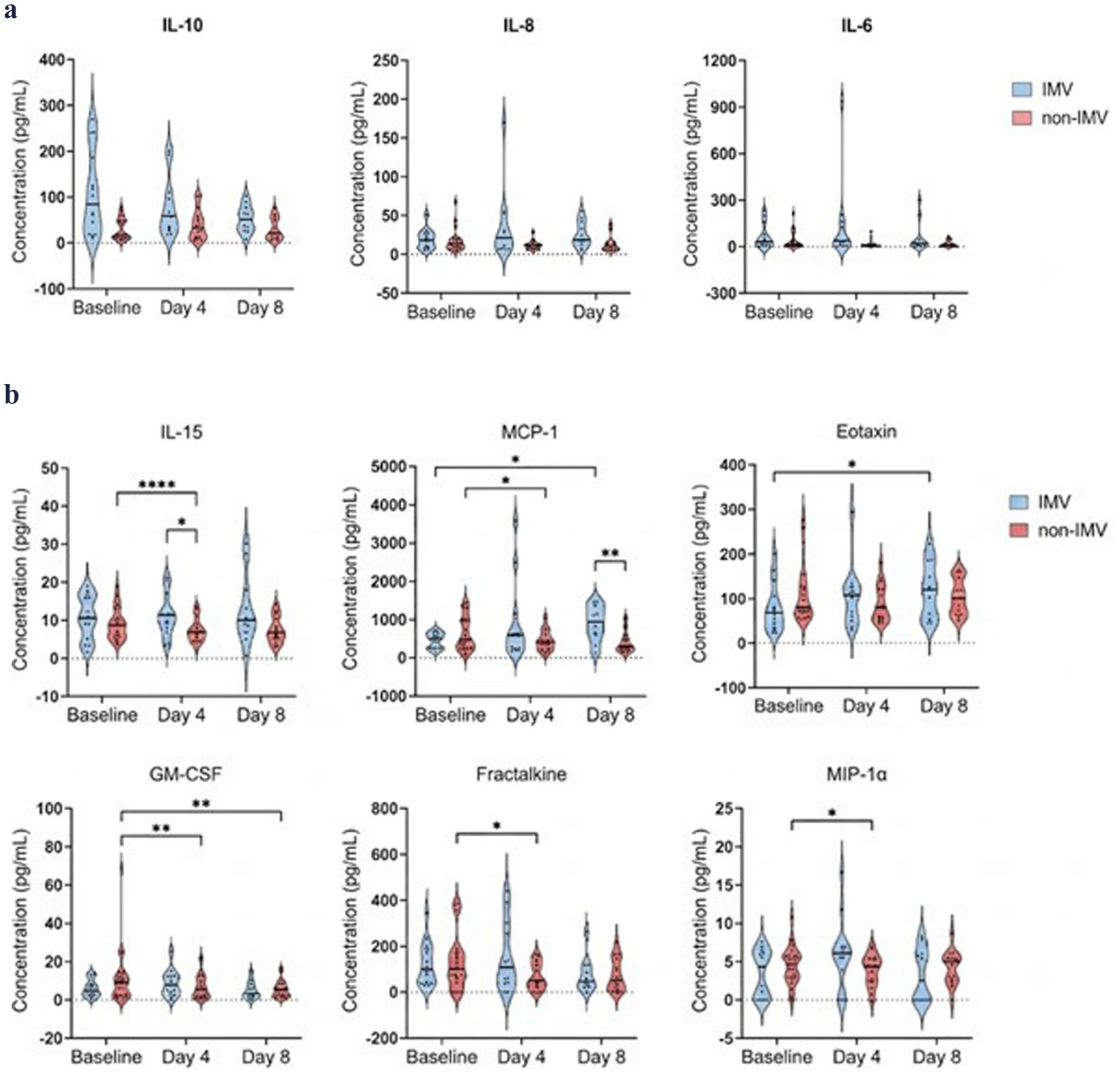
Plasma cytokine profiles in COVID-19 ARDS patients requiring IMV versus non-IMV. **(a)** Plasma concentrations of IL-10, IL-8, and IL-6 were significantly higher in IMV group compared with non-IMV group across study time points. **(b)** Significant group × time interactions were observed for IL-15, MCP-1, eotaxin, GM-CSF, fractalkine, and MIP-1α. *Post hoc* analyses revealed elevated IL-15 at Day 8 and higher MCP-1 at Day 4 in IMV group relative to non-IMV group. *p < 0.05, ** *p* < 0.01, **** *p* < 0.0005.

**Figure 4:**
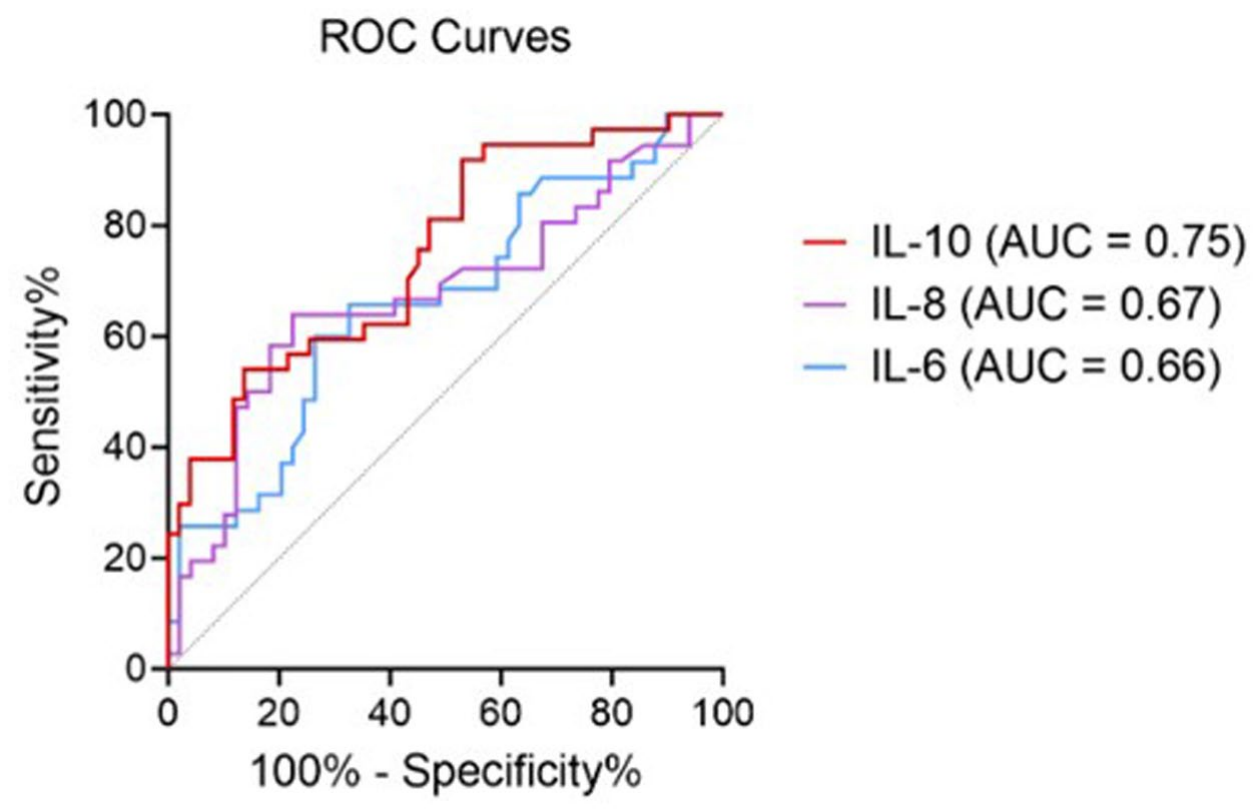
Receiver operating characteristic (ROC) curve analysis of plasma cytokines in COVID-19 ARDS patients. ROC curves illustrate the discriminatory performance of IL-10 (red), IL-8 (purple), and IL-6 (blue) for predicting invasive mechanical ventilation (IMV).

Because transcriptomics highlighted dysregulation of IL-10 and other inflammatory mediators, we next evaluated whether these changes were mirrored at the protein level. Plasma cytokine analysis revealed significantly higher levels of IL-10, IL-8, and IL-6 in patients on IMV compared with non-IMV (Figure 3a). A significant interaction effect between patient group and time point was observed for IL-15, MCP-1, eotaxin, GM-CSF, fractalkine, and MIP-1α (Figure 3b). *Post hoc* analyses revealed that the IMV group had significantly elevated IL-15 at Day 8 and higher MCP-1 at Day 4 compared to the non-IMV group. To assess diagnostic potential, receiver operating characteristic (ROC) analyses were performed for IL-10, IL-8, and IL-6 (Figure 4). All three cytokines demonstrated statistically significant AUC values, with IL-10 showing the strongest discrimination (AUC = 0.74, 95% CI 0.63–0.86, *p* = 0.0002), followed by IL-8 and IL-6. Additional cytokines such as IP-10, MCP-1 and MIP-1*β* also showed discriminatory potential, though less robust than IL-10 (Table 6). Collectively, these results indicate that patients on IMV had higher IL-10 levels than 75% of the non-IMV patients, highlighting IL-10 as the most promising biomarker. Flt-3L, IL-17A, and TGF-*α* were excluded from analysis because more than 50% of values were below assay detection limits.

**Table 6:** Area under the curve (AUC) from ROC analysis of promising cytokines and chemokines.

Cytokine	Area	Std. Error	95% CI	*p* value
IL-10	0.7440	0.05725	0.6318 to 0.8562	0.0002
IP-10	0.6680	0.05990	0.5506 to 0.7854	0.0093
MCP-1	0.6476	0.06256	0.5250 to 0.7702	0.0250
IL-8	0.6641	0.06722	0.5324 to 0.7958	0.0135
MIP-1*β*	0.6612	0.06208	0.5395 to 0.7829	0.0149

### 3.3 NETosis analysis

We investigated whether the cytokine elevations resulting in systemic inflammation translated into enhanced neutrophil activation. However, analysis of patient plasma revealed no significant differences in MPO–DNA complex formation between IMV and non-IMV groups. In addition, NETosis was not detected in neutrophils incubated with plasma from either healthy controls or COVID-19 patients at different disease stages (Supplementary Figures 2a,b).

### 3.4 Immune cell phenotype analysis

**Figure 5:**
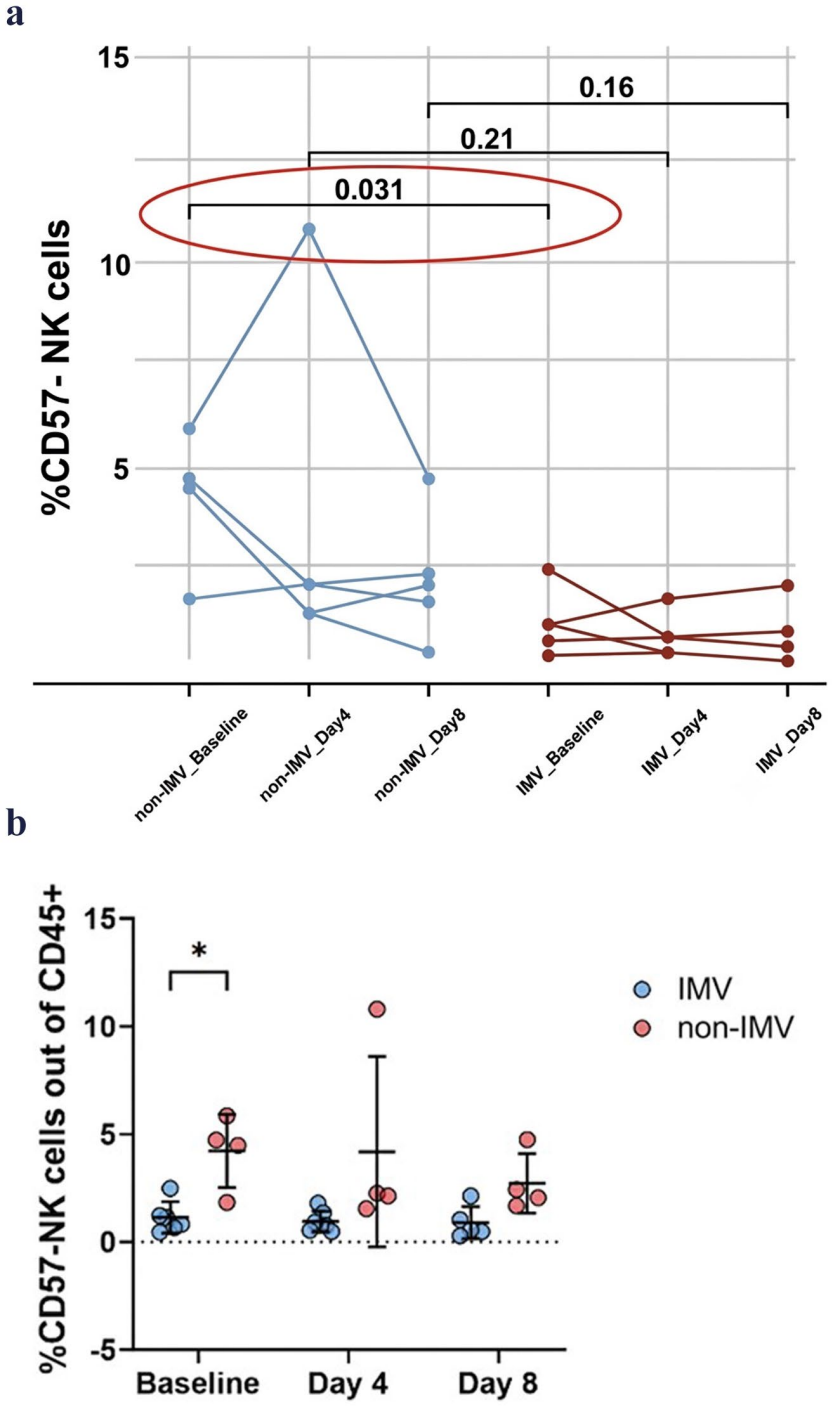
Immune cell phenotype analysis of CD57^−^ NK cells in patients on IMV versus non-IMV. **(a)** Representative cytometry by time-of-flight (CyTOF) plots showing distribution of CD57^−^ NK cells at baseline in patients requiring IMV compared with non-IMV group. **(b)** Quantitative analysis of CD57^−^ NK cell frequencies within the CD45^+^ compartment at baseline. Non-IMV group demonstrated a significantly higher proportion of CD57^−^ NK cells compared with IMV group, suggesting reduced NK cell senescence and preserved proliferative capacity in the non-IMV group. *p < 0.05.

We next performed immune cell phenotype analysis to evaluate differences in immune cell phenotypes by analyzing the proportion of CD45 + cells across immune cell subtypes. At baseline, non-IMV group had significantly higher percent of CD57^−^ natural killer (NK) cells compared with IMV group (Figures 5a,b). Since CD57 expression is a marker of NK cell senescence and reduced proliferative capacity, these findings suggest that patients on IMV may harbor a more senescent NK cell compartment, potentially impairing effective antiviral immunity and contributing to worse outcomes.

## 4 Discussion

In this study, we identified transcriptomic and immunological determinants of COVID-19 severity by comparing patients who required IMV with those managed without IMV. Our findings suggest the presence of altered mitochondrial signatures, dysregulated coagulation, cytokine imbalance, and immune exhaustion that may collectively drive progression to severe ARDS requiring invasive mechanical ventilation, consistent with emerging evidence implicating convergent immunometabolic dysfunction as a defining feature of critical COVID-19 (14–16). However, these observations should be interpreted cautiously, as the analyses were exploratory in nature, based on PBMCs, and do not establish causality or tissue-specific mechanisms underlying respiratory failure.

Transcriptomic analysis revealed a dynamic signature of disease progression. The number of differentially expressed genes was minimal at baseline, peaked on Day 4, and declined by Day 8, suggesting that systemic transcriptional dysregulation intensifies during early clinical deterioration. Profound downregulation of MTARC2, a gene that encodes a molybdenum enzyme, involved in drug metabolism and N-reductive activity, and its disruption impairs lipid and energy metabolism, suggesting a role in cellular energy regulation (17–19). The mechanism underlying reduced MTARC2 expression in PBMCs of IMV group remains unclear; however, one possibility is that impaired energy regulation, reflecting cellular hypoxemia particularly in respiratory muscles, may limit the ability to sustain spontaneous ventilation and require invasive support. Importantly, in a prior study showed that in COVID-19, the virus suppresses key mitochondrial genes needed for energy production and keeps them suppressed in critical organs like the heart, kidney, and liver, even after the virus clearance, highlighting a SARS-CoV-2-specific pattern of mitochondrial dysregulation (14). Concurrently, stress-response and ion-channel genes (RNF165, KCNMA1, AC245014.3) were upregulated, and may indicate maladaptive signaling networks in mitochondrial homeostasis and immune regulation. Of particular relevance, KCNMA1 encodes mitochondrial BK channels that regulate ROS handling under hypoxic stress, while RNF165, a RING-domain E3 ubiquitin ligase, has been shown to enhance BMP-Smad1/5/8 signaling in motor neurons through ubiquitin-mediated degradation of pathway inhibitors. Disruption of BK channel function may disturb the firing patterns of brainstem respiratory centers (such as the pre-Bötzinger complex, the key rhythm generator located in the medulla), impairing central respiratory rhythm generation and potentially contributing to respiratory failure (20). RNF165 was (16, 21) among the most consistently upregulated genes in IMV patients at both Day 4 and Day 8. Notably, RNF165-null mice exhibit shortening of phrenic nerve presynaptic branches within the diaphragm alongside evidence of impaired breathing, suggesting that dysregulated RNF165-mediated BMP signaling in phrenic motor neurons may compromise neuromuscular transmission to the diaphragm and contribute to ventilatory pump failure requiring invasive mechanical ventilation (16). The upregulation of AC245014.3, though of unknown function, may represent an unrecognized host–virus interaction. These findings position mitochondria not as passive injury targets but as active mediators of immunometabolic dysfunction.

Among the most significantly downregulated genes was TFPI, a regulator of the TF/FVIIa/FXa complex, limiting thrombin generation and exerting anti-inflammatory effects (22). Loss of TFPI has been linked to coagulopathy and mortality in COVID-19, consistent with our findings and with reports by Al-Tamimi et al. (23). This finding contrasts with systemic TFPI elevations in plasma reported during COVID-19 and may suggest compartment-specific transcriptional exhaustion under sustained inflammation (24). This paradox underscores a mitochondria–coagulation axis in which bioenergetic failure compromises vascular homeostasis and predisposes to thrombosis.

Pathway enrichment analyses further identified vascular injury and inflammatory signaling. KEGG identified enrichment of renin secretion and Rap1 signaling in patients on IMV, consistent with prior transcriptomic studies implicating dysregulation of the renin–angiotensin system and altered Rap1 signaling in severe COVID-19 (25, 26). These pathways may contribute to endothelial dysfunction, inflammation, and thrombosis associated with ARDS progression, however, these associations remain exploratory and should be validated in larger studies incorporating tissue-specific and functional analyses.

Cytokine profiling revealed elevated IL-10, IL-6, and IL-8 in patients on IMV, with IL-10 emerging as the strongest prognostic marker (AUC 0.74). While classically anti-inflammatory, IL-10 is significantly increased in patients with COVID-19 ARDS compared to those without ARDS, and this elevation is linked to disease severity, risk of pulmonary fibrosis, and adverse outcomes (27, 28). This paradoxical elevation reflects a failing compensatory mechanism or monocyte resistance to IL-10 with downstream T- and NK-cell hyperactivation. Taken together, IL-10 serves both as a marker of immune dysregulation and as a prognostic biomarker of disease severity.

A notable discrepancy emerged with IL-10 expression. Multiplex immunoassay revealed higher plasma IL-10 levels in IMV group, yet PBMC RNA-seq demonstrated downregulation of IL-10 transcripts. This likely reflects cellular composition, as IL-10 is predominantly secreted by monocytes and macrophages, which are underrepresented in PBMC isolates. Plasma IL-10 thus represents systemic monocyte/macrophage activation, while PBMC transcriptional profiles primarily capture T- and B-cell compartments.

Interestingly, we did not observe significant differences in NET formation between IMV and non-IMV groups. This contrasts with prior studies that associated excessive NET release with severe COVID-19, ARDS, and thromboinflammation (29), such as those by Melero et al. (30). Potential explanations include differences in patient populations, timing of sample acquisition, clinical management (e.g., anticoagulation use), and methodological limitations in NET quantification. While our results diverge from prior reports, they should be interpreted cautiously given the modest sample size, and they do not diminish the established role of NETs in COVID-19 pathophysiology.

Our immune phenotyping results extend these observations. Patients in the non-IMV group had a higher percentage of CD57-negative NK cells at baseline, representing a less terminally differentiated, more proliferative subset capable of expansion and functional response during viral infection. Patients requiring IMV, by contrast, exhibited enrichment of CD57-positive NK cells, consistent with a terminally differentiated phenotype characterized by reduced proliferative capacity and altered cytokine responsiveness, which may reflect impaired capacity for adaptive NK cell expansion against SARS-CoV-2. These findings align with studies by Zheng et al. and Varchetta et al., which described NK cell depletion, functional exhaustion, and skewing toward terminally differentiated subsets in severe COVID-19 (31, 32). Although these findings suggest that baseline NK cell differentiation status may be associated with disease severity and progression to respiratory failure requiring IMV, larger prospective studies are needed to determine their prognostic value and potential therapeutic implications.

### 4.1 Limitations

This study has a few limitations. First, there was a significant difference in corticosteroid exposure between the IMV and non-IMV groups, with the non-IMV cohort receiving higher daily steroid doses. Because corticosteroids are known to exert broad immunomodulatory effects, including suppression of inflammatory signaling pathways and alteration of gene transcription profiles, it is possible that some of the observed immune exhaustion signatures and transcriptomic differences were influenced, at least in part, by differences in steroid exposure rather than exclusively reflecting disease severity. Importantly, however, patients requiring IMV demonstrated evidence of immune dysregulation despite receiving lower mean steroid doses, suggesting that the observed alterations are unlikely to be solely attributable to corticosteroid therapy. Nevertheless, we recognize steroid use as a potential confounding factor that may have affected both immune phenotypes and gene expression patterns. Given the limited sample size, formal multivariable adjustment was constrained; however, this issue should be interpreted carefully when considering the biological significance of our findings. Future studies with larger cohorts and standardized corticosteroid protocols will be important to better distinguish the independent contributions of disease severity and steroid exposure to the immune and transcriptomic changes observed in critically ill patients. Second, A limitation of this study is the relatively small sample size across the different analytical platforms, including RNA sequencing, CyTOF, and cytokine profiling. The number of samples analyzed for each technique is reported in the corresponding methods. Although the longitudinal and multi-omics design provided important mechanistic insights, the limited cohort size may reduce statistical power, increase susceptibility to type I and type II errors, and raise the possibility of overfitting in multivariable analyses. Therefore, the findings should be interpreted cautiously and considered hypothesis-generating. Larger, independent validation cohorts will be necessary to confirm the robustness and generalizability of the observed immune and transcriptomic signatures. A major limitation of this study is that the transcriptomic and immunophenotyping analyses were performed using peripheral blood mononuclear cells (PBMCs) rather than tissue-derived samples from the lung, respiratory musculature, or central nervous system. As a result, the observed molecular signatures reflect systemic peripheral immune responses and may not fully represent tissue-specific mechanisms driving respiratory failure or ARDS progression in COVID-19. Future studies integrating tissue-based analyses, single-cell approaches, and functional validation experiments will be necessary to define the mechanistic relevance of these pathways in severe COVID-19 and the need for invasive mechanical ventilation.

### 4.2 Interpretation

In summary, this study identifies mitochondrial and immune dysfunction as defining molecular features of severe COVID-19–associated ARDS. First, mitochondrial gene expression patterns, along with plasma IL-10 levels and NK cell phenotypes, may serve as biomarkers to stratify patients at risk of respiratory failure and IMV. Second, therapeutic strategies aimed at restoring mitochondrial function whether by enhancing biogenesis, reducing oxidative stress, or modulating cytokine-driven metabolic shifts could address the convergent mechanisms of coagulopathy, immune exhaustion, and respiratory decompensation. Finally, the identification of TFPI dysregulation suggests that targeted anticoagulant strategies should account for mitochondrial-driven endothelial dysfunction. A deeper understanding of the mechanisms driving respiratory decompensation may ultimately improve risk stratification, guide clinical decision-making, and enhance patient outcomes.

## Data Availability

The data that support the findings of this study have been deposited in the NCBI Gene Expression Omnibus (GEO) repository and are publicly accessible at https://www.ncbi.nlm.nih.gov/geo/ under accession number (GSE336577). The original transcriptomic contributions presented in this study are publicly available. Further inquiries can be directed to the corresponding author.

## Supplementary material

SUPPLEMENTARY FIGURE 1 **(a)** Gene ontology analysis of significant DEGs in the PBMCs of intubated patients compared with non-intubated patients at day 4 and day 8 using g:Profiler. The significantly changed terms enriched at day 4 by GO molecular function (GO:MF), GO biological process (GO:BP), GO cellular component (GO:CC), KEGG, Reactome (REAC), Wikipathways (WP) and Human phenotype ontology (HP) databases. **(b)** KEGG pathway analysis via ShinyGO for significant DEGs at day 4. **(c)** Summary of enrichment analysis in DisGeNET at Day 4. There is no significantly changed terms enriched at day 8 for Gene ontology analysis.

SUPPLEMENTARY FIGURE 2 **(a)** PMA-stimulate netosis in healthy control. Green indicates dying/dead cells, some cells showed larger area of green objects can be identified as netosis cells. **(b)** Netosis in COVID-19 subject. Netosis cells based on the area filter of min 345um2 were segmented in purple.

## Glossary

AUC: area under the curve
ARDS: acute respiratory distress syndrome
BMI: body mass index
CCI: Charlson Comorbidity Index
CyTOF: cytometry by time-of-flight
DEG: differentially expressed gene
DisGeNET: database of gene–disease associations
EGF: epidermal growth factor
ELISA: enzyme-linked immunosorbent assay
FGF-2: fibroblast growth factor 2
Flt-3L: FMS-like tyrosine kinase 3 ligand
G-CSF: granulocyte colony-stimulating factor
GM-CSF: granulocyte-macrophage colony-stimulating factor
GO: gene ontology
GO:BP: gene ontology: biological process
GO:CC: gene ontology: cellular component
GO:MF: gene ontology: molecular function
GRO: growth-regulated oncogene
IFN: interferon
IFN-*α*2: interferon alpha 2
IFN-*γ*: interferon gamma
IL: interleukin
IL-1RA: interleukin-1 receptor antagonist
IP-10: interferon-gamma–inducible protein 10
IQR: interquartile range
jvenn: JavaScript Venn diagram tool
KEGG: Kyoto Encyclopedia of Genes and Genomes
MCP: monocyte chemoattractant protein
MDC: macrophage-derived chemokine
MIP: macrophage inflammatory protein
MPO: myeloperoxidase
NETs: neutrophil extracellular traps
PBMC: peripheral blood mononuclear cell
qPCR: quantitative polymerase chain reaction
RNA-seq: RNA sequencing
ROC: receiver operating characteristic
sCD40L: soluble CD40 ligand
SD: standard deviation
SCS: set counts and sizes (g:SCS threshold, g:Profiler)
TGF-α: transforming growth factor alpha
TNF: tumor necrosis factor
VEGF: vascular endothelial growth factor
WHO: World Health Organization
