# Supplementary figures and images for: Integrated transcriptomic and immune analysis reveals distinct mitochondrial and immune signature in COVID-19 ARDS requiring invasive mechanical ventilation
Source: Front Med (Lausanne). 2026 Jul 8;13:1856132. doi: 10.3389/fmed.2026.1856132 (PMC13388564; doi:10.3389/fmed.2026.1856132)

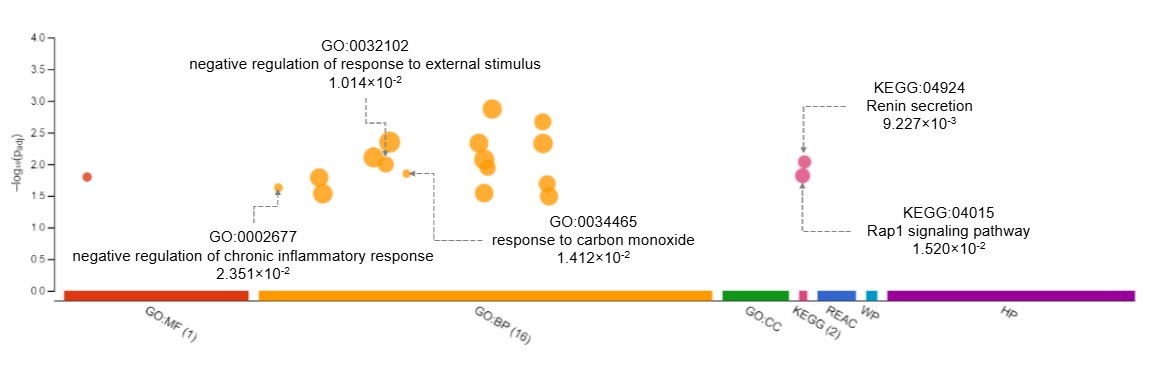

Supplement: SUPPLEMENTARY FIGURE 1 — (a) Gene ontology analysis of significant DEGs in the PBMCs of intubated patients compared with non-intubated patients at day 4 and day 8 using g:Profiler. The significantly changed terms enriched at day 4 by GO molecular function (GO:MF), GO biological process (GO:BP), GO cellular component (GO:CC), KEGG, Reactome (REAC), Wikipathways (WP) and Human phenotype ontology (HP) databases. (b) KEGG pathway analysis via ShinyGO for significant DEGs at day 4. (c) Summary of enrichment analysis in DisGeNET at Day 4. There is no significantly changed terms enriched at day 8 for Gene ontology analysis. [file Image_1.JPEG]

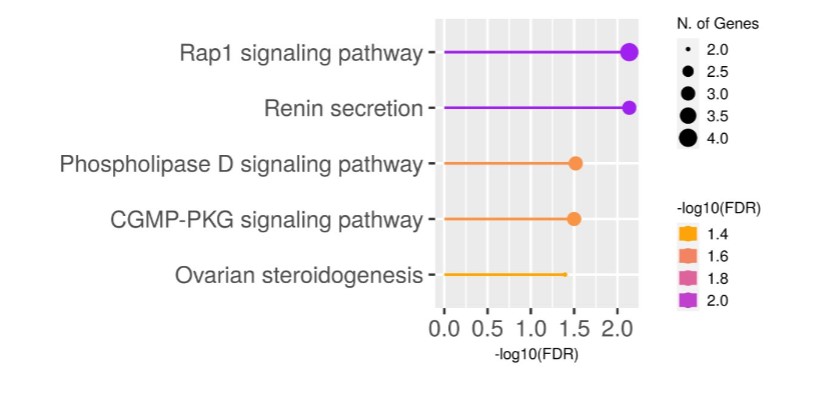

Supplement: SUPPLEMENTARY FIGURE 2 — (a) PMA-stimulate netosis in healthy control. Green indicates dying/dead cells, some cells showed larger area of green objects can be identified as netosis cells. (b) Netosis in COVID-19 subject. Netosis cells based on the area filter of min 345um2 were segmented in purple. [file Image_2.JPEG]

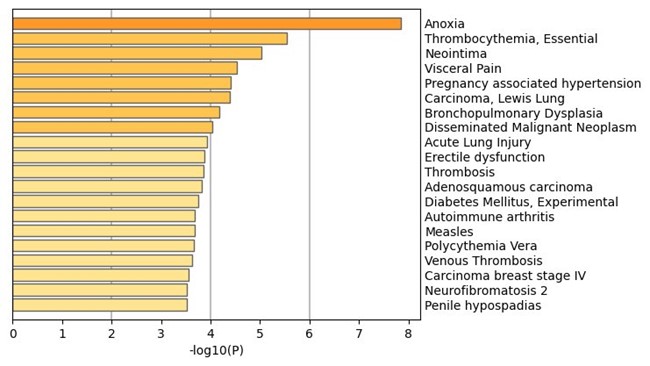

Supplement: Supplementary file 3 [file Image_3.JPEG]

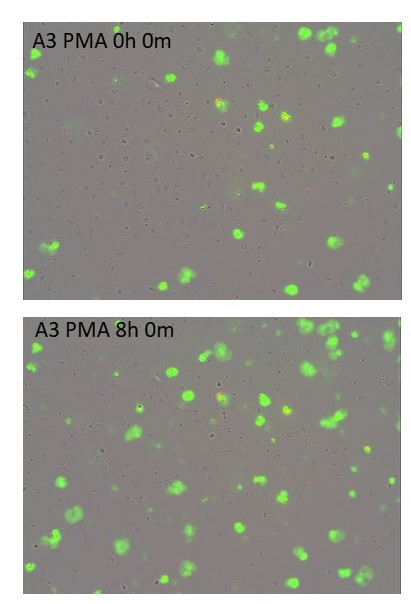

Supplement: Supplementary file 4 [file Image_4.JPEG]

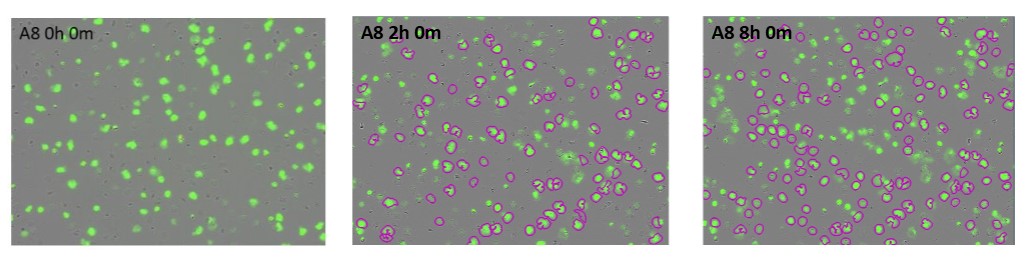

Supplement: Supplementary file 5 [file Image_5.JPEG]
